# Rituximab in pediatric refractory nephrotic syndrome: a systematic review and meta-analysis evaluating therapeutic efficacy and adverse event profiles

**DOI:** 10.1007/s00467-025-07013-8

**Published:** 2025-11-08

**Authors:** Junchao Deng, Lizhen Zhu, Xiaoshi Zhu

**Affiliations:** 1 Department of Pediatrics, Sichuan Academy of Medical Sciences and Sichuan People’s Hospital, No.32 West Section 2, First Ring Road, Qingyang District, Chengdu, Sichuan 610072 People’s Republic of China; 2 Emergency Care Unit, Sichuan Academy of Medical Sciences and Sichuan People’s Hospital, Chengdu, Sichuan 610072 People’s Republic of China

**Keywords:** Immunosuppressant, Rituximab, Refractory nephrotic syndrome, Pediatric, Meta-analysis

## Abstract

**Background:**

Rituximab is considered a therapeutic option in children with refractory nephrotic syndrome (RNS). However, the efficacy and safety of rituximab treatment remain unclear.

**Objective:**

This study systematically evaluated the clinical effectiveness and related adverse event profiles of rituximab compared with other immunosuppressants in children with RNS.

**Method:**

Two independent investigators conducted the methodological rigor evaluation in a double-blind manner. Statistical analyses were performed using RevMan 5.4.

**Results:**

Relative to other conventional second-line agents, rituximab therapy presented a significantly increased relapse-free survival rate [vs. tacrolimus: OR = 3.42, 95% CI = 1.96–5.94, *P* < 0.01]. Fewer adverse drug reactions occurred in patients with rituximab than with other immunosuppressants [vs. cyclophosphamide: OR = 0.18, 95% CI = 0.04–0.89, *P* = 0.03; vs. tacrolimus: OR = 0.26, 95% CI = 0.07–0.92, *P* = 0.04]. The application of rituximab increased the steroid-discontinued rate and decreased the corticosteroid dose in the treatment [vs. cyclophosphamide: OR = 5.46, 95% CI = 2.00–14.92, *P* = 0.0009; vs. tacrolimus: OR = 3.42, 95% CI = 1.67–7.01, *P* = 0.0008].

**Conclusion:**

Our study demonstrates rituximab’s superior efficacy and safety profile compared to conventional second-line immunosuppressants in pediatric RNS, particularly regarding steroid minimization. Systematic review registration number: CRD420251082051.

**Graphical Abstract:**

**Figure:**
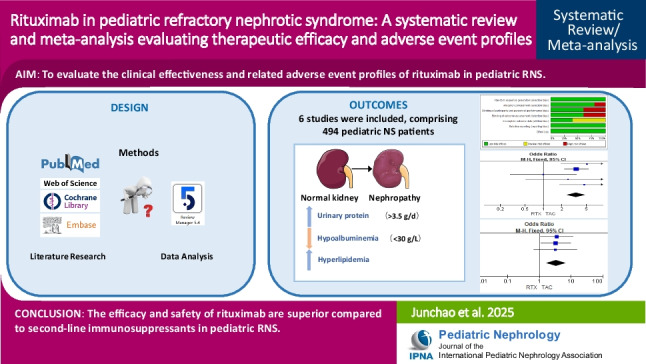
A higher resolution version of the Graphical abstract is available as Supplementary information

**Supplementary information:**

The online version contains supplementary material available at 10.1007/s00467-025-07013-8.

## Introduction

Pediatric primary nephrotic syndrome (PNS) is distinguished by the clinical triad of massive proteinuria (urinary protein excretion > 3.5 g/d), hypoalbuminemia (serum albumin < 30 g/L), hyperlipidemia, and peripheral edema [1]. However, 40% of these patients progress to pediatric refractory nephrotic syndrome (RNS) [2], encompassing frequently-relapsing NS (FRNS), steroid-dependent NS (SDNS) and steroid-resistant NS (SRNS). While glucocorticoids (GC) remain first-line therapy per KDIGO guidelines [3], chronic exposure leads to debilitating complications in > 80% of cases, including cushingoid features, growth retardation, and opportunistic infections.

Current management strategies emphasize steroid-sparing protocols using second-line agents: cyclophosphamide (CPM) demonstrates 45%−60% sustained remission but carries dose-dependent gonadotoxicity risk [4]; calcineurin inhibitors (CNIs) like tacrolimus achieve 65−75% remission rates yet incur nephrotoxicity in 38% of patients [5]. Rituximab (RTX) specifically targets B cells by binding to CD20 and depletes them, with emerging evidence suggesting a superior relapse prevention [6]. Recent cohort studies report 72% 2-year relapse-free survival with RTX vs. 54% with CNIs, alongside a 63% reduction in annual steroid exposure.

Despite accumulating observational data, critical knowledge gaps persist. First, direct comparisons between RTX and conventional immunosuppressants remain limited to small single-center studies with heterogeneous outcome measures. Second, optimal dosing regimens (fixed-dose vs. B-cell guided protocols) lack consensus, particularly in weight-stratified pediatric populations. Third, long-term safety profiles beyond 5-year follow-up are inadequately characterized, especially regarding hypogammaglobulinemia and vaccine responses.

RTX and other second-line immunosuppressive agents are the common treatments for pediatric RNS. Their efficacy and safety have been heavily reported. At present, the most appropriate second-line therapy has not been identified for children with RNS. Therefore, we undertook a meta-analysis to compare the efficacy of pediatric RNS between RTX and common second-line drugs, aiming to inform evidence-based selection of second-line therapies in pediatric nephrology practice.

## Methods

### Literature search

The articles were searched from the following online databases (up to December 2024): PubMed, Embase, Web of Science and Cochrane Library. We retrieved articles importing the combination of MeSH terms and related text-word terms, including “rituximab”, “cyclophosphamide”, “cyclosporine”, “tacrolimus”, “mycophenolate mofetil”, “nephrotic syndrome”, “children”, and “pediatric”.

## Inclusion criteria

- Type of research: Randomized controlled trials (RCT), cohort studies and prospective longitudinal investigations.
- Population: Children with RNS ≤ 18 years old.
- Intervention and comparator: Rituximab vs. cyclophosphamide, rituximab vs. cyclosporine A, rituximab vs. tacrolimus, and rituximab vs. mycophenolate mofetil.
- Outcome: Relapse-free survival rate, adverse events and corticosteroid dose.

## Exclusion criteria

Articles not written in English, case reports, studies with incomplete data and repeat studies.

### Article and data extraction

A dual-reviewer methodology was rigorously implemented across two sequential screening phases: initial eligibility assessment based on titles and abstracts, followed by full-text evaluation against predefined inclusion criteria. Extracted parameters included the first author, publication year, study design, cohort size, intervention (dosage, administration, and course of treatment) and outcomes. Findings from the screening process were summarized using a flow diagram.

### Risk of bias assessment

The methodological quality and potential biases of the literature were independently assessed by two investigators using the Review Manager software (RevMan 5.4 RRID: SCR_003581). The bias assessment domains are as follows: (1) random sequence generation and allocation concealment; (2) blinding of participants and personnel; (3) blinding of outcome assessment; (4) incomplete outcome data; (5) selective reporting; and (6) other bias. The risk level of each indicator was represented by “low risk of bias”, “unclear risk of bias”, or “high risk of bias”.

### Statistical analysis

The primary outcome was relapse-free survival rate (proportion of patients without recurrence or associated complications leading to death during the follow-up period). The secondary endpoint encompassed safety evaluation (adverse events) and quantitative assessment of total cumulative glucocorticoid administration.

Data were analyzed by RevMan 5.4 software. The association between intervention and relapse-free survival was quantified using odd ratios (OR) with 95% confidence intervals (CI); the incidence of adverse events was also compared between groups using OR or risk ratios (RR) with corresponding 95% CI. Heterogeneity analysis was conducted by *Q* test and *I*^*2*^ test. The random-effects model was used to combine the effect size when heterogeneity was substantial (*I*^*2*^ > 50% and *P* < 0.05), whereas a fixed-effect model was applied under homogeneity assumptions (*I*^*2*^ < 50% and *P* ≥ 0.05). A *P*-value < 0.05 suggested a statistically significant difference between groups. Forest plots were generated to visually synthesize meta-analytic estimates across included studies, while the potential publication bias was assessed by funnel plots. A tow-tailed *P* < 0.05 threshold defined statistical significance. To evaluate the robustness and validate the reproducibility of conclusions, sensitivity analyses were performed using a leave-one-out methodology.

### Subgroup analysis

To further assess the efficacy and safety of RTX in the treatment of RNS in children, subgroup analyses were performed based on disease phenotypes, dividing children into FRNS/SDNS and SSNS groups.

## Results

### Study characteristics

**Fig. 1:**
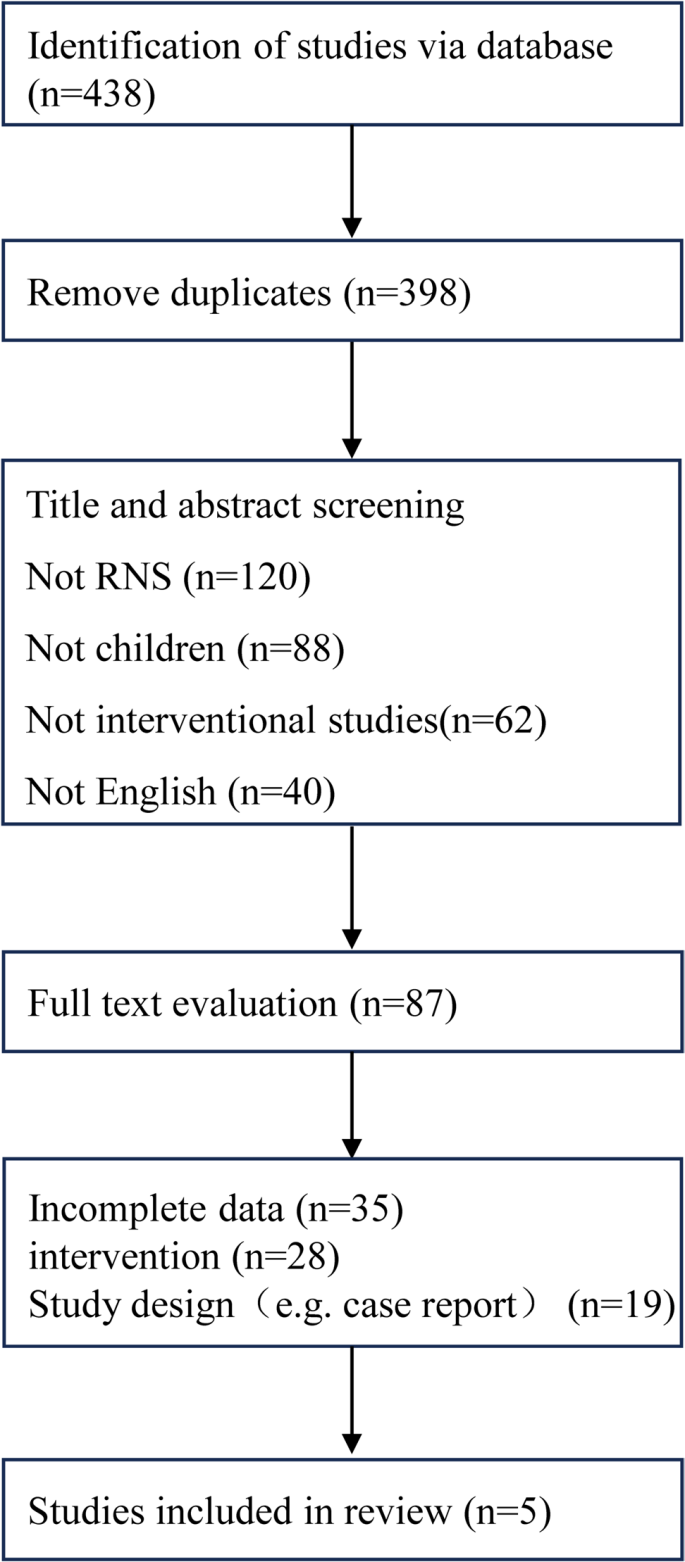
PRISMA flow diagram of the literature research

Our systematic search identified 438 potential studies across designated databases. Following duplicate removal (*n* = 40), 398 records underwent title/abstract screening utilizing predefined eligibility criteria. Of these, 87 articles progressed to full-text assessment, culminating in the inclusion of 5 randomized controlled trials for final analysis [7–11] (see PRISMA flowchart in Fig. 1).

The pooled cohort comprised 494 pediatric participants with refractory nephrotic syndrome, stratified into four treatment arms:

1. Rituximab with glucocorticoids (*n* = 145; median dose 375 mg/m^2^ × 2 cycles).
2. Cyclophosphamide with corticosteroids (*n* = 44; cumulative dose 168 ± 25 mg/kg).
3. Tacrolimus combined with steroid therapy (*n* = 155; trough 5–10 ng/mL).

Summary of study details, such as research design, cohort size, intervention, course of treatment, and outcome, are systematically tabulated in Table 1.

**Table 1:** Study and patient characteristics of included studies

Parameter	Type of research	Enrolled patients	Sample size		Intervention		Course of treatment	Outcome	
			experimental	control	experimental	control		Primary outcome	Secondary outcome
Kari 2020 [1]	Open label randomized study	FR/SDNS	19	27	RTX, 375 mg/m^2^, 2 dose, interval of 2 weeks	CPM, 3 mg/kg/d, 8 weeks	12 months	Relapse-free survival rate	Adverse events
Wang 2022 [2]	RCT	FR/SDNS	17	17	RTX, 375 mg/m^2^,	CPM, 10 mg/kg/d, 2days/w	12 months	Relapse-free survival rate	Relapse, adverse events, prednisolone dosage, HRQOL
Wang 2022^#^[2]	RCT	FR/SDNS	17	17	RTX, 375 mg/m^2^	TAC, 0.1–0.15 mg/kg/d, 2 dose, first 6 months	12 months	Relapse-free survival rate	Relapse, adverse events, prednisolone dosage, HRQOL
Basu 2018 [3]	RCT	SDNS	60	60	RTX, 375 mg/m^2^, 2 dose	TAC, 0.2 mg/kg/d	12 months	Relapse-free survival rate	Relapse, cumulative corticosteroid dose, adverse events
Mathew 2022 [4]	Open label RCT	SSNS	20	20	RTX, 375 mg/m^2^, 2 dose	TAC, 0.1–0.2 mg/kg/d	12 months	Proportion of patients with sustained remission	Frequent relapses, treatment failure, cumulative prednisolone dose, adverse events
Solomon 2019 [5]	Open label randomized study	SDNS	59	58	RTX, 375 mg/m^2^, 2–4 dose	TAC, 0.1–0.2 mg/kg/d	12 months	Relapse-free survival rate	Relapse rate, cumulative prednisolone dosage, adverse events, steroid-free rate

Wang L 2022^#^: This study compared rituximab with different immunosuppressive agents

*RCT* randomized controlled trials, *FRNS* frequently-relapsing nephrotic syndrome, *SDNS* steroid-dependent nephrotic syndrome, *SSNS* steroid-sensitve nephrotic syndrome, *RTX* rituximab, *CPM* cyclophosphamide, *TAC* tacrolimus, *HRQOL* Health-Related Quality of Life

### Risk of bias

**Fig. 2:**
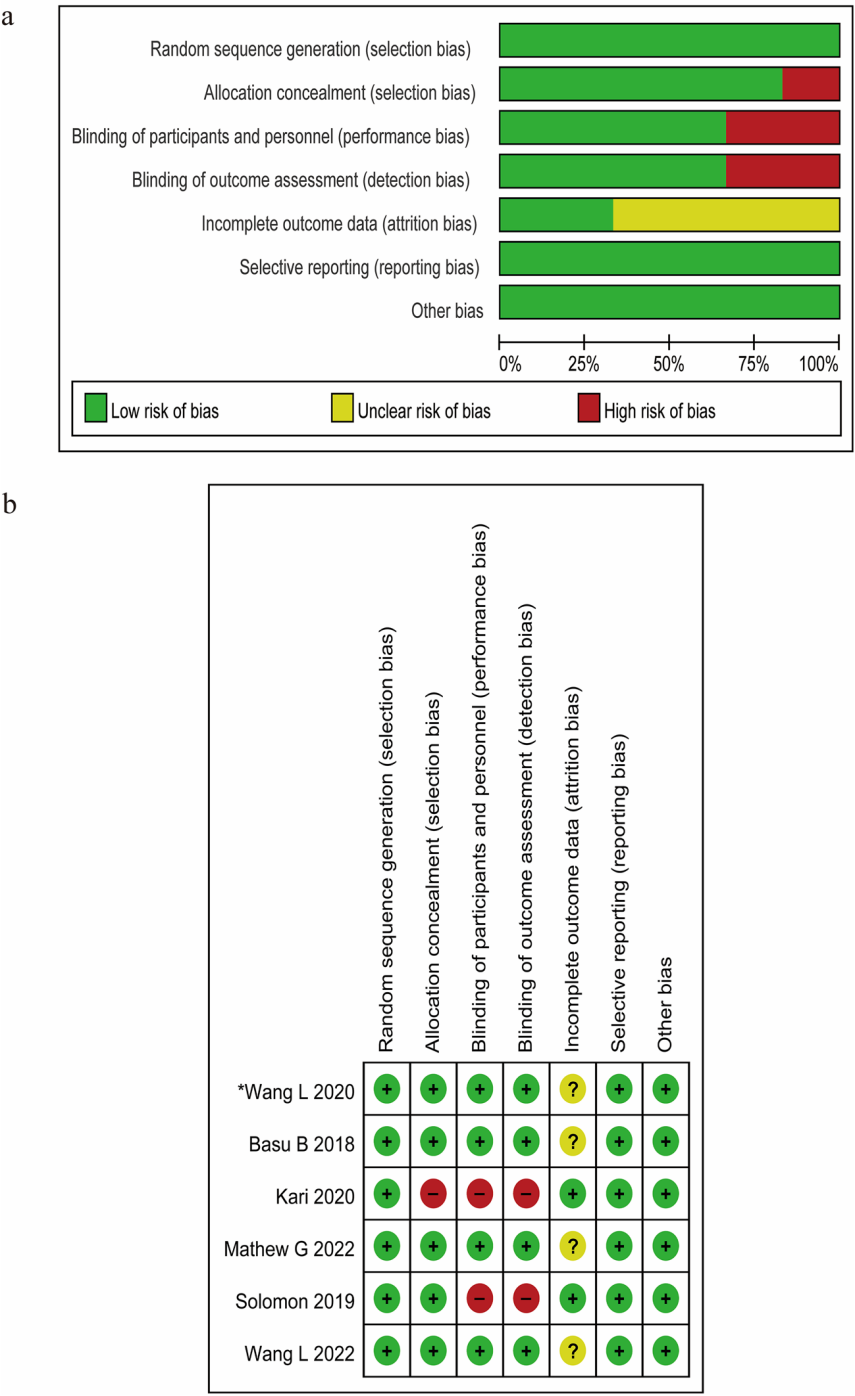
Risk of bias: assessment of risk of bias for all studies. **a** Risk of bias graph. **b** Risk of bias summary

Results of the methodological quality assessment of all included studies are summarized graphically in Fig. 2. All studies demonstrated low risk of bias (green, “ + ”) in critical domains, including random sequence generation and allocation concealment, except the study by Kari et al. (red, “ + ”) [8]. The majority of studies employed a double-blind design; however, the blinding was not implemented in the trial by Kari et al. [8] and Solomon et al. [10], resulting in a high risk (red, “-”). Three studies [7, 9, 11] exhibited unclear risk of bias (yellow, “?”) due to incomplete data from participant withdrawal or loss to follow-up. Additionally, one trial demonstrated unclear risk of bias in selective reporting (yellow, “?”).

### Primary outcome: relapse prevention

**Fig. 3:**
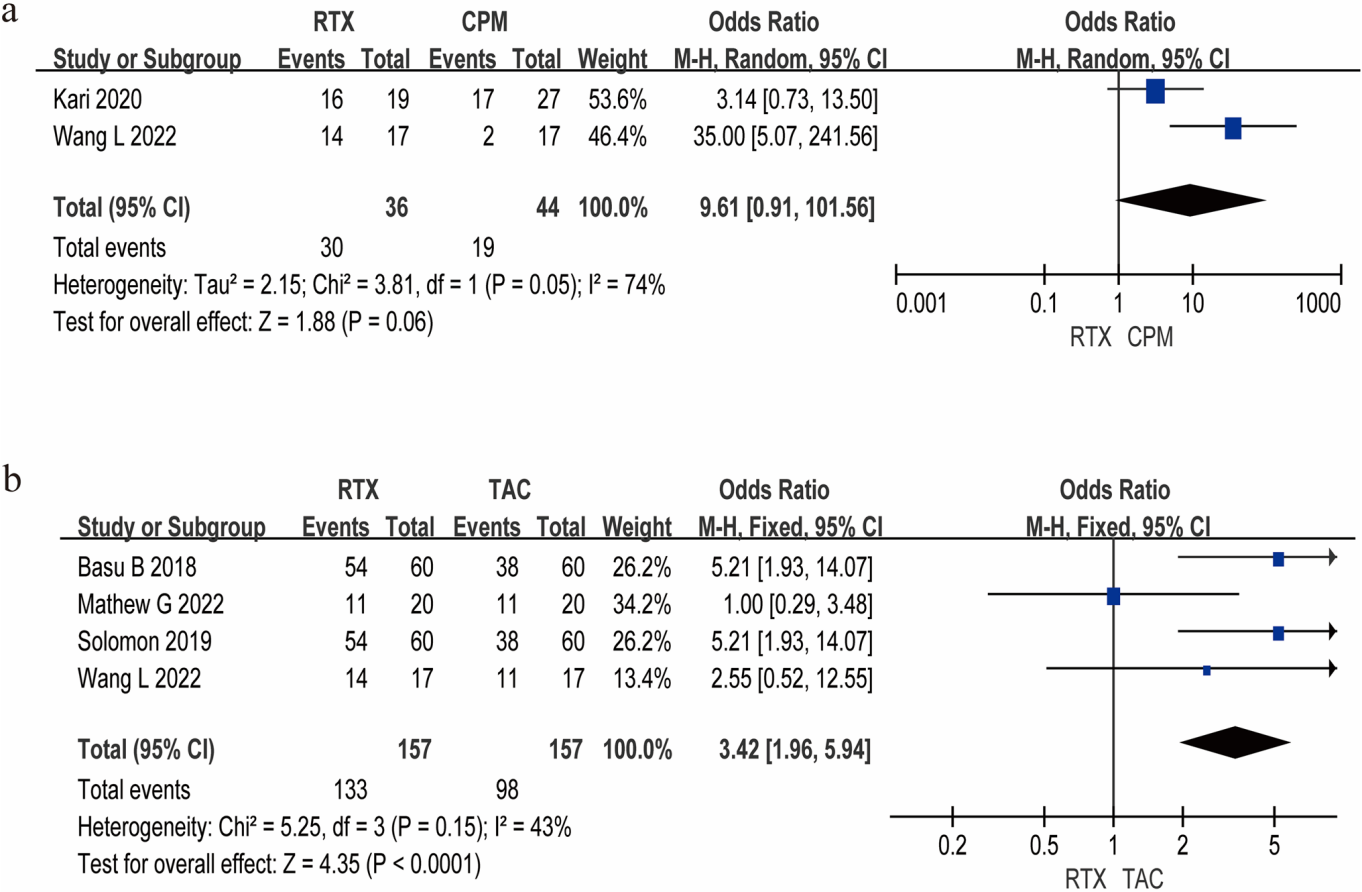
Meta-analysis results of efficacy for RTX in RNS. **a** Comparative analysis of relapse-free survival between RTX and CPM. **b** Relapse-free survival outcomes for RTX versus TAC

The meta-analysis demonstrated significant differential efficacy profiles among second-line therapies. RTX showed a clinically meaningful trend vs. CPM in relapse prevention (OR = 9.61, 95% CI 0.91–101.56, *P* = 0.06), and substantial heterogeneity emerged (*I*^*2*^ = 74%, *P* = 0.05), likely attributable to protocol variations in CPM administration and cumulative dosing ranges (Fig. 3a). Notably, RTX exhibited superior relapse prevention over tacrolimus (TAC) with a 3.42-fold lower relapse risk (95% CI 1.96–5.94, *P* < 0.01), demonstrating robust consistency (*I*^*2*^ = 43%, *P* = 0.15) (Fig. 3b). This corresponded to 12-month relapse-free survival rates of 79% (95% CI 65%−93%) for RTX vs. 62% (95% CI 58%−65%) for TAC, highlighting RTX's durable disease control capacity. The funnel plot results are present in Supplementary Figure S1.

### Adverse events

**Fig. 4:**
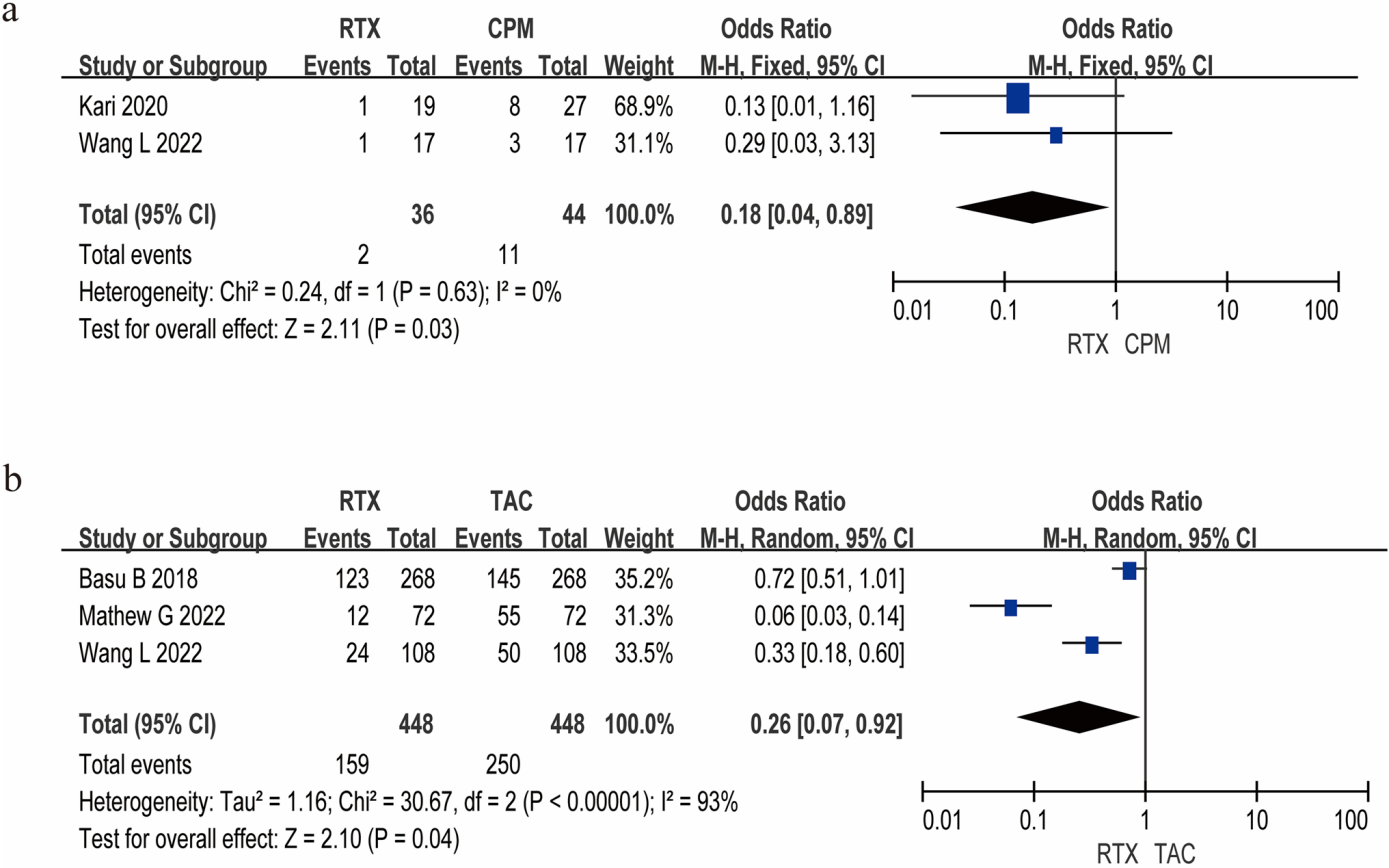
Meta-analysis results of the incidence of adverse events for RTX in RNS. **a** Adverse event incidence rate of RTX and CPM. **b** Adverse event incidence rate of RTX and TAC

Common adverse events (AEs) associated with second-line therapies for RNS encompassed severe infection, gastrointestinal reaction, reduction of kidney function, hypertension, and others. As depicted in Fig. 4, the number of patients with AEs in the RTX group was significantly lower than that in the CPM group (*P* < 0.05). Additionally, pooled analysis demonstrated significantly fewer AEs in RTX-treated patients compared to those receiving TAC (*P* < 0.05). Potential publication bias was evaluated through funnel plot analysis (Supplementary Figure S2).

### Corticosteroid usage

**Fig. 5:**
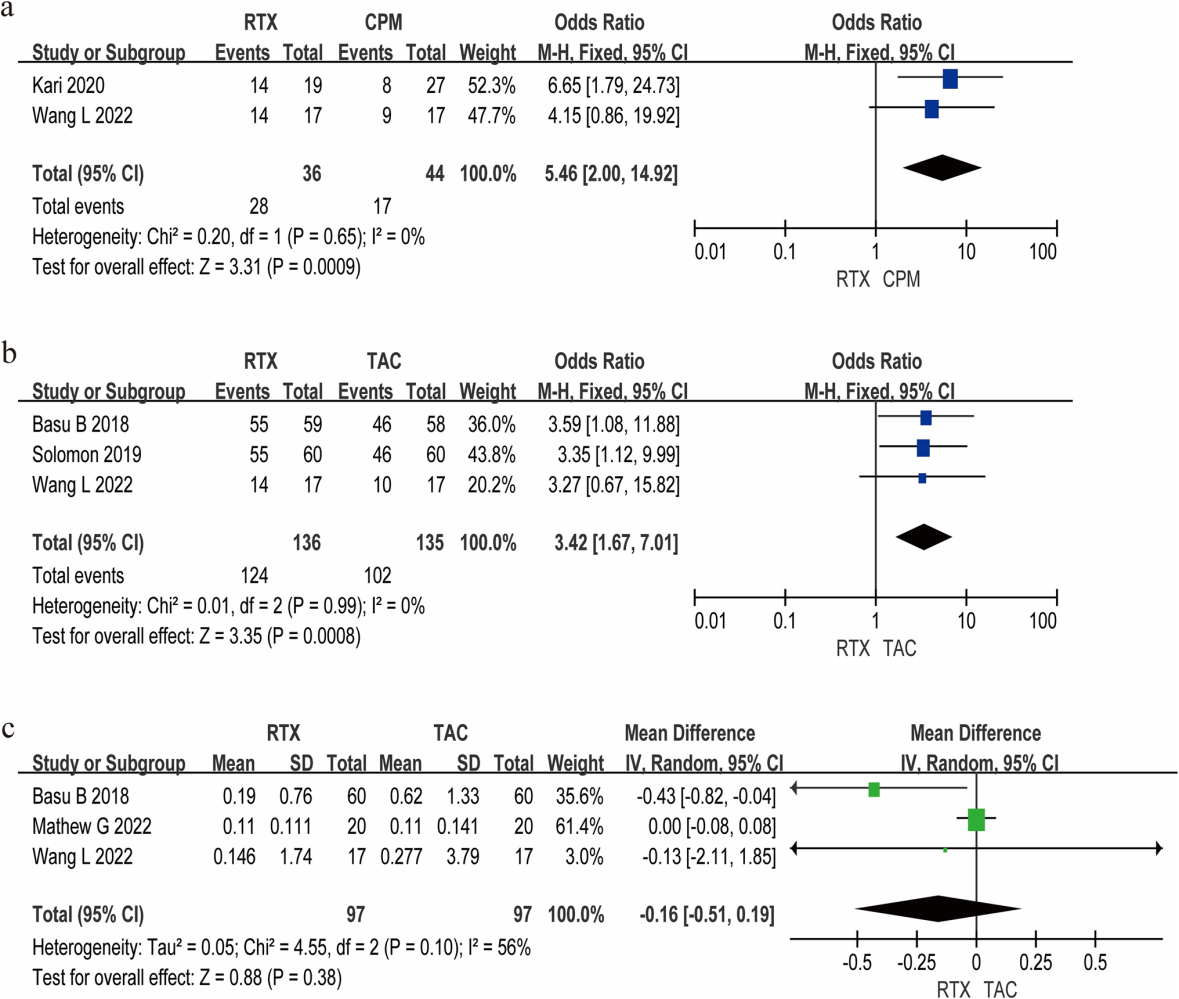
Meta-analysis results of steroid-discontinued rate and cumulative steroid dose. **a** Steroid-discontinued rate of RTX and CPM. **b** Steroid-discontinued rate of RTX and TAC. **c** Cumulative steroid dose in RTX therapy and TAC therapy

The meta-analysis revealed significant intergroup differences in glucocorticoid utilization patterns. As shown in Fig. 5, compared to TAC-based regimens, RTX therapy resulted in more patients not receiving corticosteroids with a 3.42-fold higher withdrawal rate (95% CI 1.67–7.01, *P* < 0.01), achieving a marked reduction in cumulative steroid exposure, with a mean difference of −0.16 mg/kg/d (95% CI −0.51 to 0.19, *P* = 0.38) over 12-month follow-up. Furthermore, RTX demonstrated superior steroid-sparing capacity vs. CPM, yielding 5.46-fold higher rates of complete steroid discontinuation (95% CI 2.00–14.92, *P* = 0.0009). These findings underscore RTX's dual advantage in both dose reduction and treatment-free remission attainment compared to conventional immunosuppressants. Funnel plots evaluating publication bias for corticosteroid dosage are provided in Supplementary Figure S3.

### Subgroup analysis

**Fig. 6:**
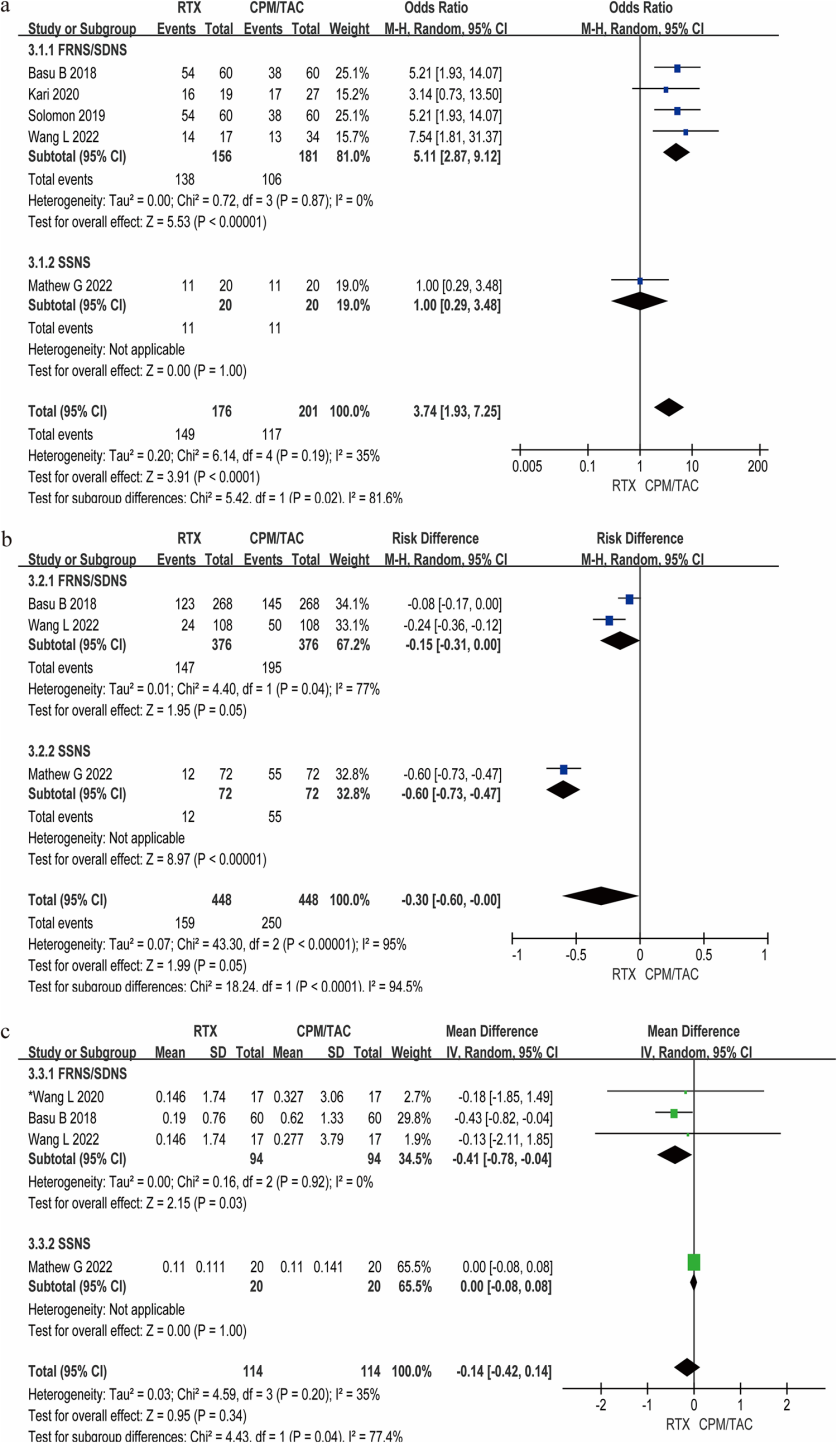
Subgroup-analysis results based on disease phenotypes. **a** Relapse-free survival rate of RTX and CPM/TAC. **b** Adverse event incidence of RTX and CPM/TAC. **c** Cumulative steroid dose in RTX therapy and CPM/TAC therapy

To explore the heterogeneity of the included population and verify the robustness of the results, subgroup analyses were performed (Fig. 6). Patients were divided into FRNS/SDNS and SSNS groups. The relapse prevention, adverse events, and corticosteroid usage were primary outcome measures. In FRNS/SDNS patients, RTX significantly increased the relapse-free survival rate compared with CPM/TAC (95% CI 2.87–9.12, P < 0.01), which confirms the superior efficacy of RTX in reducing the risk of recurrence in this specific population. In patients with SSNS, there is no difference between RTX and TAC (OR = 1, 95% CI 0.29–3.48), which may be due to the limited number of articles included and the small sample size. The overall effect indicated the superior efficacy of RTX vs. CPM/TAC, with a 3.74-fold higher relapse-free survival rate (95% CI 1.93–7.25, *P* < 0.01). The incidence of adverse events in patients receiving RTX was significantly lower than that in patients with CPM/TAC (95% CI −0.60 to 0.00, *P* < 0.01), which was consistent in both FRNS/SDNS (*P* < 0.05) and SSNS (P < 0.01) subgroups. In the FRNS/SDNS group, RTX treatment achieved a markedly lower cumulative corticosteroid dose (−0.41 kg/mg/d, 95% CI −0.78 to −0.04, *P* < 0.05), while in the SSNS group, there was no statistical significance (*P* = 1.00). Funnel plot analysis of subgroup analyses are shown in Supplementary Figure S4.

## Discussion

Three phenotypes of RNS (SRNS, SDNS, and FRNS) displayed typical manifestations of nephrotic syndrome, including massive proteinuria, edema, and hypoalbuminemia. Patients with SRNS are characterized by steroid resistance and persistent unresolved proteinuria after 4 weeks of standard treatment with prednisolone. In contrast, SDNS patients are sensitive to glucocorticoid (GC) therapy, experiencing 2 consecutive relapses (urinary protein ≥ 50 mg/kg) during the treatment course or within 14 days of discontinuation. Meanwhile, FRNS is defined by a higher frequency of recurrence, indicating ≥ 2 relapses within 6 months of initial remission or ≥ 4 relapses during any subsequent 12 months. Children with RNS usually have a poor prognosis and a risk of progressive kidney dysfunction. An estimated 5−14% of them advance to chronic kidney failure, contributing substantially to global childhood incidence and death rates [12]. Additionally, NS patients who achieve complete remission after 4 weeks of standard treatment with prednisone or prednisolone are considered as steroid-sensitive. While SSNS are not inherently classified as RNS, our inclusion of three such studies reflects the clinical reality that disease progression or suboptimal management may precipitate refractory phenotypes.

Current KDIGO guidelines prioritize glucocorticoids as first-line treatment, yet 40% of patients on GC therapy develop steroid dependence or frequently relapse [1]. Additionally, significant serious adverse events are associated in the long-term treatment with high-dose steroids, such as growth retardation, osteoporosis, metabolic complications and increased infection risk [13, 14]. These limitations underscore the imperative for safer steroid-sparing agents.

The immunopathogenesis of RNS involves complex T/B lymphocyte dysregulation culminating in podocyte injury. Calcineurin inhibitors (CNIs), including CsA and TAC, serve as guideline-recommended second-line therapy for RNS. CNIs potently improve the barrier function of the glomerulus [15]. CNIs block T lymphocyte activation and cytokine production by inhibiting calcineurin activity [16], further exerting an immunosuppressive effect and reducing the level of urinary protein. CNIs also produce nephrotoxicity and destroy kidney function while treating RNS. Only one clinical trial [17] has compared the efficacy of CsA and RTX, reporting a higher remission rate with RTX compared to CsA (150 mg/m^2^/day) in patients with SRNS, shedding light on the necessity for larger-scale controlled trials to validate these findings. CPM, an immunosuppressive alkylating agent widely used in the clinical management of PNS, exerts its therapeutic effects by damaging the structure and function of DNA, selectively inhibiting the proliferation and differentiation of lymphocytes, especially B-cell populations critical to autoimmune pathogenesis [18, 19]. Nevertheless, the clinical utility of CPM is substantially limited by dose-dependent nephrotoxicity, including but not limited to kidney impairment, gastrointestinal reaction, liver damage, alopecia, risk of infertility and other adverse effects [20].

Notably, mycophenolate mofetil (MMF) – an alternative second-line agent for CPM-intolerant patients – was excluded due to insufficient comparative data. The sole available trial [21] reported unexpectedly high relapse rates with MMF vs. RTX (375 mg/m^2^). Nevertheless, this observation requires validation through larger randomized controlled studies.

Considering the serious side effects of these immunosuppressants, a more efficient and safer intervention is needed for long-term treatment. Rituximab is a monoclonal antibody specifically targeting Cluster of Differentiation 20 (CD20), a membrane protein on B cells. This specific targeting triggers the depletion of B cells [6, 22]. Due to the depletion of B cells from RTX, the expression of autoantibodies (such as the antibody against nephrin) may decline, reducing the production of pathogenic IgG antibodies, thereby diminishing the formation and deposition of immune complexes along the glomerular basement membrane, primarily via suppression of antibody-producing B-cell clones. Anti-nephrin antibody has been detected in both adults and children with minimal change disease, which is the primary pathogenesis of pediatric nephrotic syndrome [23, 24]. High expression of anti-nephrin antibody is associated with post-transplant proteinuria recurrence severity. A Japanese cohort study confirmed the relationship between anti-nephrin antibody and recurrence in pediatric patients [25]. Emerging clinical evidence supports RTX as a promising intervention for RNS. An article reported that RTX intervention before transplant contributed to the significant reduction of anti-nephrin antibodies in a child with focal segmental glomerulosclerosis (FSGS) [26]. Similarly, Ranani et al. [27] reported only one relapse among children with SDNS treated with RTX, contrasting sharply with prednisone-treated controls, alongside a 42% reduction in proteinuria. For FRNS [28], RTX significantly extended the median relapse-free period compared to conventional therapies. Furthermore, steroid-resistant patients achieved sustained proteinuria remission under RTX regimens [29, 30].

Other anti-CD20 drugs like ofatumumab (OFA) and obinutuzumab (OBI) provide alternative options for RTX-resistant or allergic patients. In the study by Ravani et al. [31], a single infusion of OFA did not induce remission in multidrug-resistant NS, while in a randomized trial [32], ofatumumab was not superior to RTX in SDNS and CNIs-dependent NS patients. No comparison between RTX and OBI was conducted, but clinical trials to evaluate the efficacy of OBI reported that low-dose OBI was a potential therapeutic option for children with FRNS/SDNS [33].

Our meta-analysis corroborates these findings, demonstrating RTX's superiority in improving relapse-free survival (OR = 3.42 vs. TAC, *P* < 0.01), while reducing severe adverse events vs. TAC (*P* = 0.04) and CPM (*P* = 0.03). Critically, RTX enabled corticosteroid dose reduction (MD = −0.16 mg/kg/d, *P* = 0.38) and higher steroid withdrawal rates (OR = 5.46 vs. CPM, *P* = 0.0009; OR = 3.42 vs. TAC, *P* = 0.0008), mitigating long-term glucocorticoid toxicity. Furthermore, the subgroup analysis confirmed that RTX has superior efficacy and safety compared with CPM/TAC, while these were mainly reflected in patients with FRNS/SDNS, especially in the relapse prevention and corticosteroid discontinuation. In contrast, RTX did not express a better efficacy in SSNS patients, which may be because of the limitations of the included studies and sample size.

Prior meta-analyses [34, 35] established the therapeutic profile of RTX in refractory nephrotic syndrome, while our study specifically addresses the pediatric population and provides a direct comparison with conventional second-line immunosuppressants. Consistently, RTX significantly improved relapse-free survival and remission rates. In this article, new evidence further supports the robustness of the original conclusion. In addition, we find that compared with other second-line agents, RTX has fewer adverse events in children with RNS. Despite these advantages, during clinical application of RTX in the treatment of pediatric RNS, infusion reaction, hypogammaglobulinemia, nervous system adverse reactions, and other side effects should still be monitored [36]. Infusion-related reactions are the most common adverse events with RTX treatment, which were also present in our analysis. Although hypogammaglobulinemia is commonly observed in the use of RTX for treating FR/SDNS [37], in the patients included in the present analysis, hypogammaglobulinemia was not recorded in any infection events. Cognitive disturbance and mood alteration are primary nervous system reactions [7], but only a few cases occurred in the included children.

## Conclusions

Rituximab may be a beneficial option for pediatric RNS. This meta-analysis demonstrates that RTX exhibits superior therapeutic efficacy and safety compared to conventional immunosuppressants in pediatric RNS. However, the current evidence is limited by the number and scope of available studies, precluding a comprehensive comparison with agents like CsA or MMF. Prospective, large-scale, well-designed and high-quality multicenter RCTs focused on pediatric populations are critical for conclusively demonstrating sustained therapeutic efficacy, longitudinal safety outcomes, and risk–benefit profiles of RTX across diverse demographic subgroups with refractory nephrotic syndrome, thereby advancing the translation of clinical evidence into practice and informing evidence-driven guideline development.

## Supplementary information

Below is the link to the electronic supplementary material.

Graphical Abstract (PPTX 438 KB)

ESM2 (DOCX 237 KB)

## Data Availability

The original contributions presented in the study are included in the article and supplementary materials. Further inquiries can be directed to the corresponding authors.

## Abbreviations

NS: Nephrotic syndrome
RNS: Refractory nephrotic syndrome
PNS: Primary nephrotic syndrome
FRNS: Frequently-relapsing nephrotic syndrome
SDNS: Steroid-dependent nephrotic syndrome
SRNS: Steroid-resistant nephrotic syndrome
SSNS: Steroid-sensitive nephrotic syndrome
RCT: Randomized controlled trials
GC: Glucocorticoids
CPM: Cyclophosphamide
CNIs: Calcineurin inhibitors
RTX: Rituximab
CsA: Cyclosporine A
TAC: Tacrolimus
MMF: Mycophenolate mofetil
OFA: Ofatumumab
OBI: Obinutuzumab
AE: Adverse event
MN: Membranous nephropathy
