# Supplementary material for: Rituximab in pediatric refractory nephrotic syndrome: a systematic review and meta-analysis evaluating therapeutic efficacy and adverse event profiles
Source: Pediatr Nephrol. 2025 Nov 8;41(6):1611–22. doi: 10.1007/s00467-025-07013-8 (PMC13139277; doi:10.1007/s00467-025-07013-8)
Supplement: Supplementary file 1 — Graphical Abstract (PPTX 438 KB) [file 467_2025_7013_MOESM1_ESM.pptx]

## Slide 1
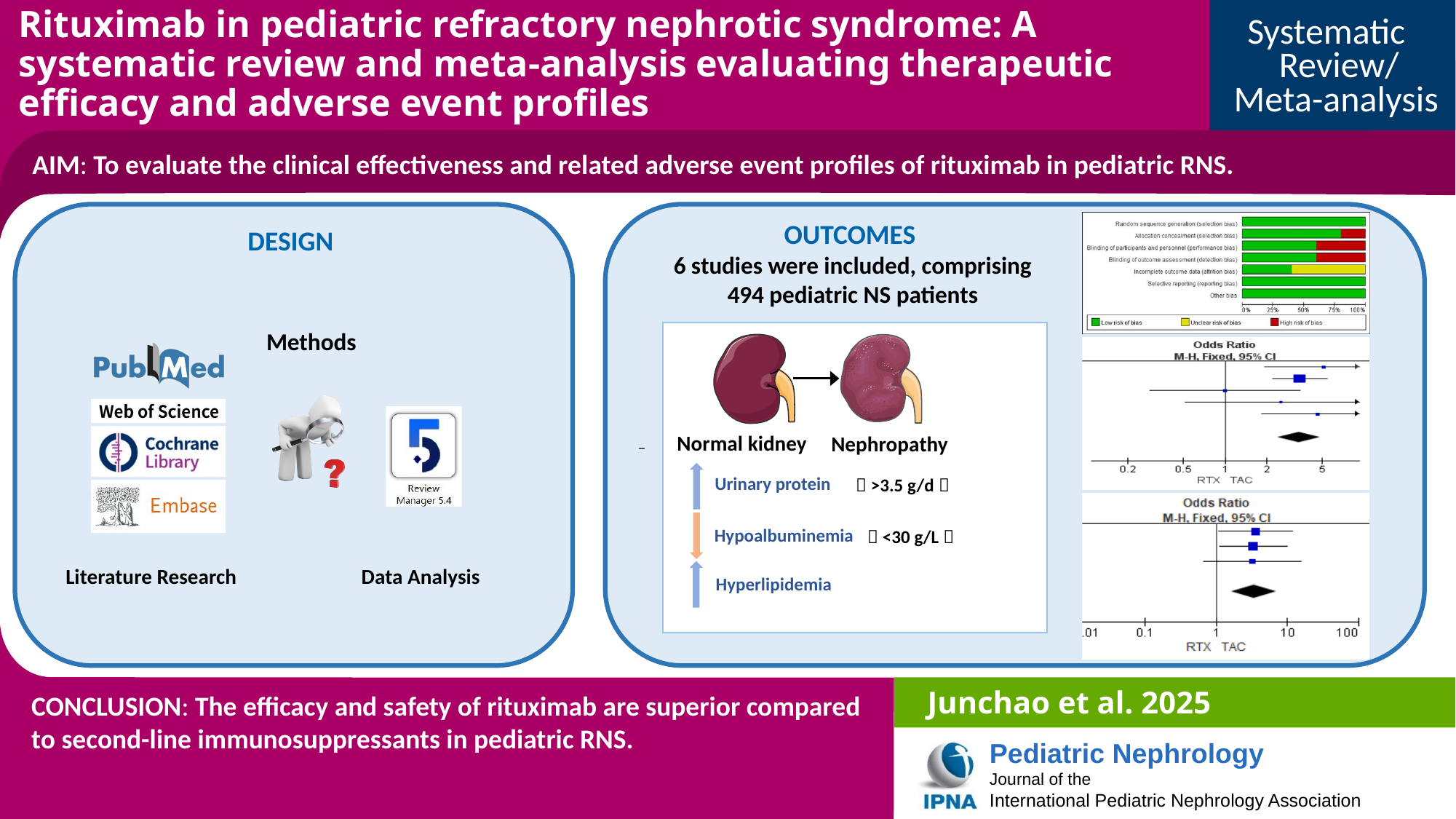

Rituximab in pediatric refractory nephrotic syndrome: A systematic review and meta-analysis evaluating therapeutic efficacy and adverse event profiles
AIM: To evaluate the clinical effectiveness and related adverse event profiles of rituximab in pediatric RNS.
OUTCOMES
6 studies were included, comprising 494 pediatric NS patients
DESIGN
Methods
Normal kidney
Nephropathy
Urinary protein
（>3.5 g/d）
Hypoalbuminemia
（<30 g/L）
Hyperlipidemia
Literature Research
Data Analysis
Junchao et al. 2025
CONCLUSION: The efficacy and safety of rituximab are superior compared to second-line immunosuppressants in pediatric RNS.
