# Supplementary material for: Rituximab in pediatric refractory nephrotic syndrome: a systematic review and meta-analysis evaluating therapeutic efficacy and adverse event profiles
Source: Pediatr Nephrol. 2025 Nov 8;41(6):1611–22. doi: 10.1007/s00467-025-07013-8 (PMC13139277; doi:10.1007/s00467-025-07013-8)
Supplement: Supplementary file 2 — (DOCX 237 KB) [file 467_2025_7013_MOESM2_ESM.docx]

**Rituximab in pediatric refractory nephrotic syndrome: A systematic review and meta-analysis evaluating therapeutic efficacy and adverse event profiles**

**Authors：**Junchao Deng^1*^, Lizhen Zhu^2^，Xiaoshi Zhu^1*^

1. Department of Pediatrics, Sichuan Academy of Medical Sciences and Sichuan People’s Hospital, Chengdu, Sichuan, 610072, PR China

2. Emergency Care Unit, Sichuan Academy of Medical Sciences and Sichuan People’s Hospital, Chengdu, Sichuan, 610072, PR, China

***Corresponding author:**

Junchao Deng, Email: [dengjunchao13@163.com](mailto:dengjunchao13@163.com)

Department of Pediatrics, Sichuan Academy of Medical Sciences and Sichuan People’s Hospital,

NO.32 West Section 2, First Ring Road, Qingyang District, Chengdu 610072, Sichuan, PR China

Xiaoshi Zhu, Email: [zhu8031@126.com](mailto:zhu8031@126.com)

Department of Pediatrics, Sichuan Academy of Medical Sciences and Sichuan People’s Hospital,

NO.32 West Section 2, First Ring Road, Qingyang District, Chengdu 610072, Sichuan, PR China


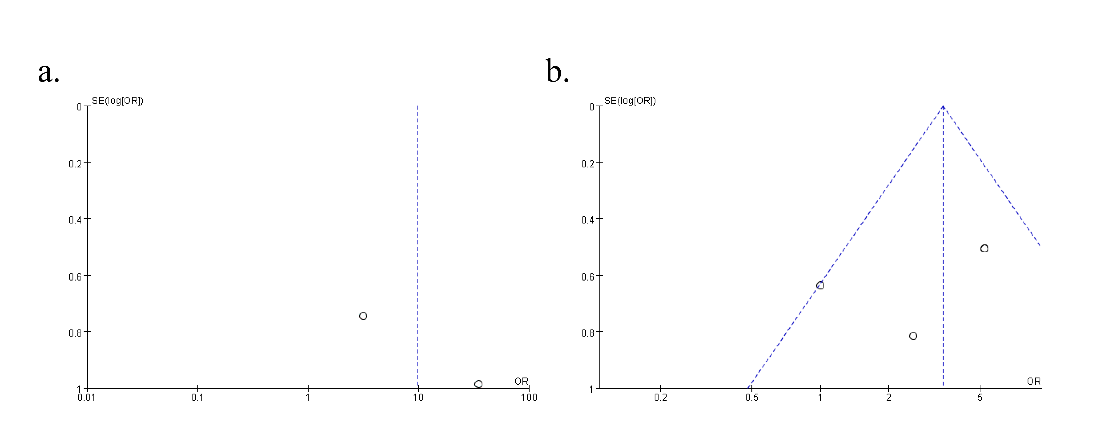


**Fig. S1** Funnel plots of remission rates and relapse-free rate by different contrasts. (a) RTX vs CPM, only two studies are included, symmetry has little significance, while combined with heterogeneous studies, the risk of bias is negligible; (b) RTX vs TAC, a missing corner in the funnel plot means that publication bias exists, which may be because one of the articles is of low quality.


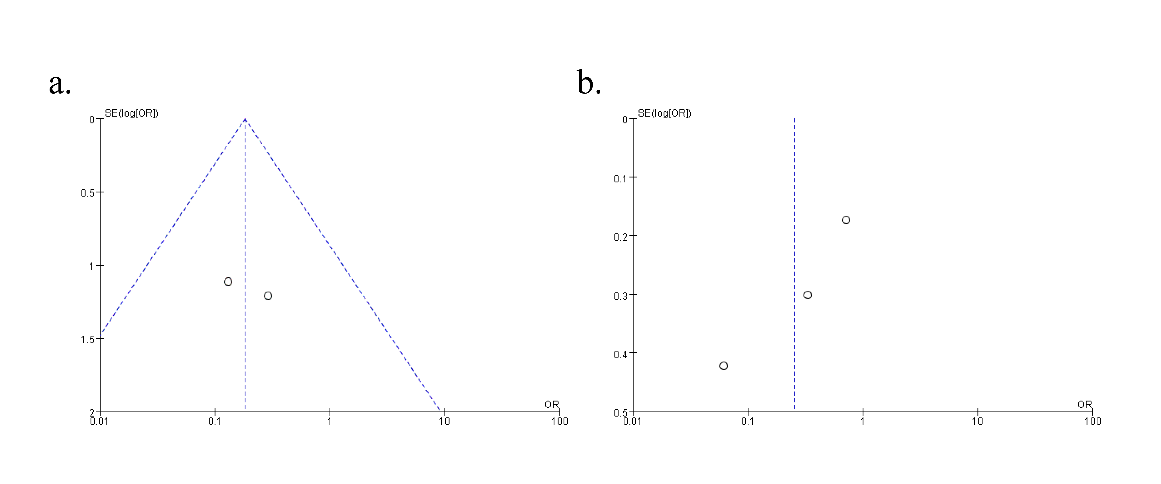


**Fig. S2** Funnel plots of adverse event incidence by different contrasts. (a) RTX vs CPM, publication bias risk is low; (b) RTX vs TAC, three circles are outside of the bias, caused by the high heterogeneity of studies.


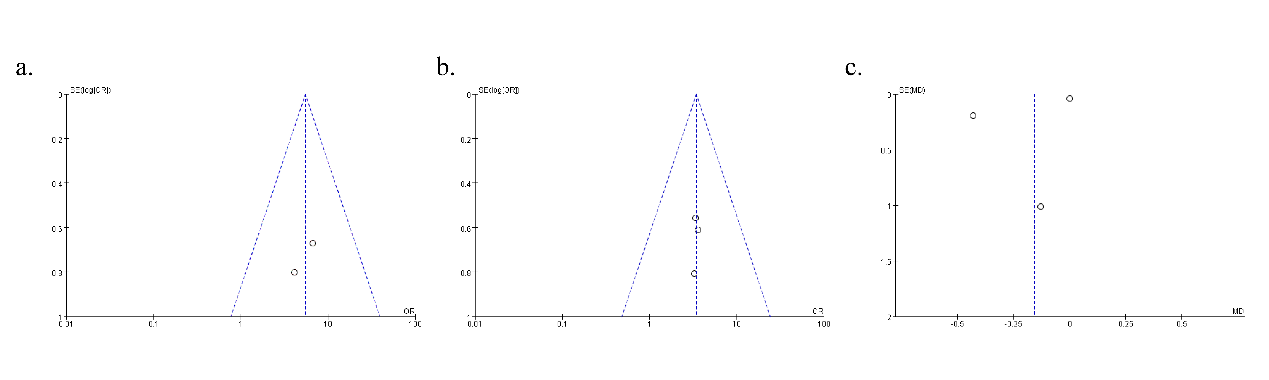


**Fig. S3** Funnel plots of steroid-discontinued rate and steroid dose by different contrasts. (a) RTX vs CPM, basically symmetrical graph means low publication bias; (b) RTX vs TAC, three studies fall near pooled effect values; (c) RTX vs TAC, not completely symmetric graph means publication bias exists, which may be caused by the heterogeneity


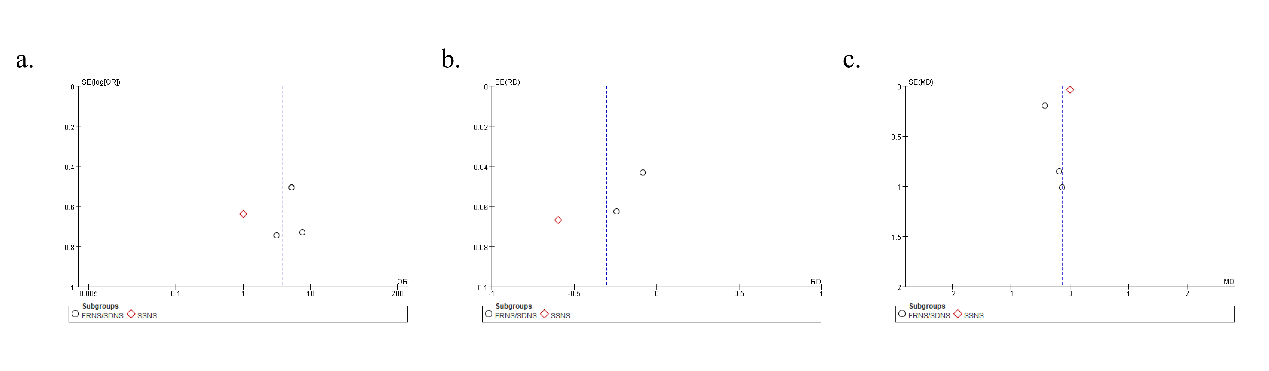


**Fig. S4** Funnel plots of subgroup analyses for RTX vs CPM/TAC. (a) relapse-free survival rate; (b) adverse events; (c) cumulative corticosteroid dosage
